# The Polygenic and Monogenic Basis of Paediatric Fractures

**DOI:** 10.1007/s11914-021-00680-0

**Published:** 2021-05-04

**Authors:** S. Ghatan, A. Costantini, R. Li, C. De Bruin, N. M. Appelman-Dijkstra, E. M. Winter, L. Oei, Carolina Medina-Gomez

**Affiliations:** 1 Translational Skeletal Genomics Group, Department of Internal Medicine, Erasmus MC University Medical Centre, Doctor Molewaterplein 40, Ee-571, 3015 GD Rotterdam, The Netherlands; 2 Department of Epidemiology, Erasmus MC University Medical Centre, Rotterdam, The Netherlands; 3 Department of Molecular Medicine and Surgery and Center for Molecular Medicine, Karolinska Institutet, Stockholm, Sweden; 4 Department of Paediatrics, Leiden University Medical Centre, Leiden, The Netherlands; 5 Department of Internal Medicine, Leiden University Medical Centre, Leiden, The Netherlands

**Keywords:** Genetics, Children, Paediatric, Fracture risk, Osteoporosis, Genome-wide association studies

## Abstract

**Purpose of Review:**

Fractures are frequently encountered in paediatric practice. Although recurrent fractures in children usually unveil a monogenic syndrome, paediatric fracture risk could be shaped by the individual genetic background influencing the acquisition of bone mineral density, and therefore, the skeletal fragility as shown in adults. Here, we examine paediatric fractures from the perspective of monogenic and complex trait genetics.

**Recent Findings:**

Large-scale genome-wide studies in children have identified ~44 genetic loci associated with fracture or bone traits whereas ~35 monogenic diseases characterized by paediatric fractures have been described.

**Summary:**

Genetic variation can predispose to paediatric fractures through monogenic risk variants with a large effect and polygenic risk involving many variants of small effects. Studying genetic factors influencing peak bone attainment might help in identifying individuals at higher risk of developing early-onset osteoporosis and discovering drug targets to be used as bone restorative pharmacotherapies to prevent, or even reverse, bone loss later in life.

## Introduction

Bone fractures are common in childhood. Among healthy children, as many as one-half of boys and one-third of girls will sustain a fracture by age 18 [1]. Most paediatric fractures are a consequence of trauma and are highly correlated with sports participation, despite the association between exercise (physical loading) and increased bone density and strength in children [2]. However, some children experience fractures as the result of underlying pathology, not trauma. Fracture incidence is dependent on many demographic factors, such as an individual’s risk-taking behaviour, nutritional state, physical activity and genetic background [3]. Genome-wide association studies (GWASs) and more advanced techniques leveraging individual genetic data, such as Mendelian randomization (MR), have demonstrated that in adults, the individual risk of fracture is determined to a great extent by the genetic variation of bone mineral density (BMD) [4]. Extrapolating these results to children would mean that the genetic underpinning of normal variation in paediatric fracture risk could comprise hundreds or thousands of genetic variants (or polymorphisms). Yet, the presence of repeated fractures in an individual may indicate an underlying bone fragility condition caused by a single gene defect. In this review, we summarize the current knowledge on the genetic architecture of paediatric fracture risk from the perspective of monogenic and complex trait genetics. We also discuss the potential of investigating fracture-related phenotypes in children, highlighting the opportunities afforded by larger collaborations and novel phenotypes.

## Fracture Risk Across the Lifespan

In general, fractures occur at an increased rate during childhood and adolescence [5, 6], then decrease in subsequent years only to rise again with increasing fragility in the elderly [7]. Overall, a fracture incidence rate of 133/10,000 person-years has been reported in an 11-year follow-up study in British children [6]. Surprisingly, the difference in fracture incidence rate between populations of younger and older age is not pronounced. For instance, participants of the Rotterdam Study aged 55 years and older present a fracture incidence rate of 189/10,000 person-years (over 20 years of follow-up) [8] which is comparable to that observed in children. The high fracture frequency in children likely reflects the vulnerability of the growing skeleton before the attainment of peak bone mass (i.e. the maximum bone mineral content and density reached between the second and third decades of life) and/or the increased fall risk brought upon physical active individuals. Fractures can occur at any stage of life but are often only considered a serious health complication when they occur in old age. This sentiment is supported by the fact that fragility fractures limit mobility and result in a high burden to individuals and their families, with some, like those of the hip, significantly increasing mortality risk during the first year after fracture [9]. In contrast, fractures that occur during childhood are not typically considered serious adverse health events, yet, when they are, it can be difficult to determine when they merit further clinical investigation. The initial assessment of the aetiology of recurring fractures in children consists of systematically ruling out a wide range of differential diagnoses, including accidental trauma, child abuse, metabolic bone disease and several other secondary causes [10].

Several reports have confirmed that children who sustain fractures have lower BMD and this parameter has been directly associated with the force of trauma applied to the bone [11–13]. In parallel, adult studies have shown that a single BMD measurement can predict fracture risk over 20–25 years [14].

Peak bone mass has been proposed as the single most important factor that can be intervened to prevent osteoporosis later in life [15]. This is expected, as peak BMD explains considerably more variance than bone loss, for any BMD measurement during late adulthood [15]. A simulation study predicted that an increase of 10% in the magnitude of peak bone mass can delay the onset of osteoporosis by 13 years [16]. This is in line with the knowledge we have gathered from GWASs showing that most of the genetic variants influencing BMD in elderly adults exert their effect early in life [17]. Thus, individuals who have a high genetic susceptibility to suffer fractures from a young age carry this risk into later life. Although environmental factors play an important role, hereditary factors contribute between 40 and 80% of the variability in peak bone mass [18, 19].

## Polygenic Basis of Paediatric Fracture

GWASs aiming to identify genetic determinants of fractures in children are rare. A recent study involving 3230 children from a cohort in Finland identified an association between one single nucleotide polymorphism (SNP) on chromosome 10 (rs112635931 minor allele frequency (MAF) = 0.05) and fractures during childhood [20]. This association signal was mapped to 10p14 in close proximity to *PROSER2* and *PROSER2-AS1*. However, replication in other paediatric cohorts to validate this finding is still needed, especially since this locus has not been reported as associated with BMD, or fracture in larger studies in children or adults [4, 17, 21, 22]. Identifying only one locus associated with paediatric fracture is not surprising considering the heterogeneity of the trait; the largest fracture GWAS in adults (~184,000 cases and ~380,000 controls) identified only 15 associated loci [4]. A more successful approach to discover the genetic underpinnings of paediatric fracture would be to investigate related relevant traits that underlie fracture risk. BMD has been shown to account for around 80% of the variation in bone strength [23] and, as stated above, is tightly related to the risk of fracture throughout life [13].

**Fig. 1:**
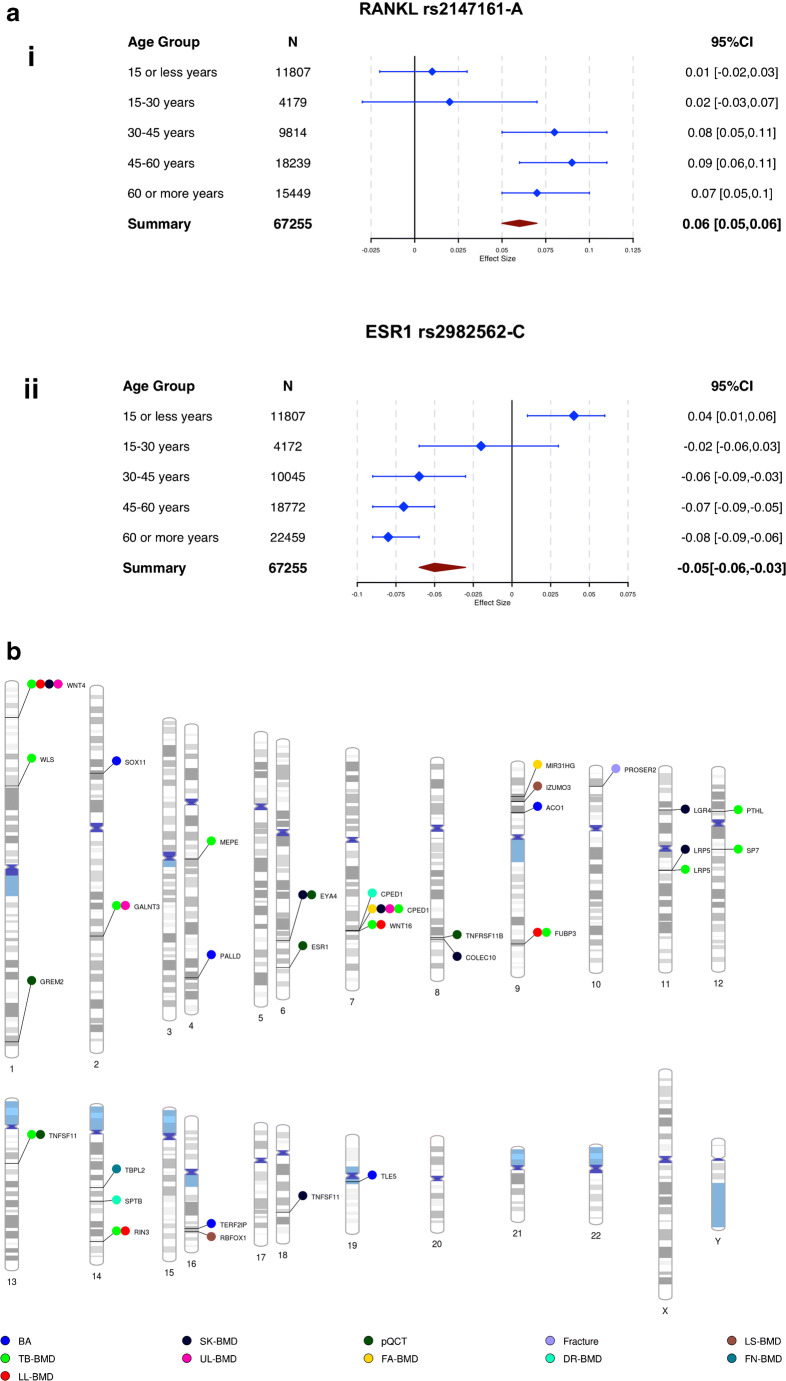
Differential estimates on bone mineral density (BMD) across the lifespan by age strata. **a** (i) The A-allele of rs2147161 SNP mapping to *RANKL*. Data from GWAS for total body bone mineral density (TB BMD). (ii) The C-allele of rs2982562 SNP mapping to *ESR1* [17]. **b** Phenogram of bone-related loci identified by GWAS in the paediatric population, mentioned in this study. The loci are named according to the closest gene, or, a biologically relevant gene within proximity. Loci relate to the following studies: TB BMD, total body BMD [17]; BA, bone accrual [29]; FA BMD, forearm BMD [27]; fracture, paediatric fractures [20]; LL BMD, lower limb BMD [25]; UL BMD, upper limb BMD [25]; SK BMD, skull BMD [25]; FN BMD, femoral neck [31]; LS BMD, lumbar spine [31]; DR BMD, distal radius BMD [31]; pQCT, peripheral quantitative computed tomography [33]

In 2009, a BMD GWAS combined samples of children and adults to identify *SP7*, encoding Osterix, an osteoblast transcription factor [24]. Since then, several studies have provided evidence that genetic factors account for a substantial proportion of the variance in paediatric BMD [17, 25–32]. To date, the largest GWAS meta-analysis of paediatric BMD comprised ~11800 children aged 3–15 years and identified 8 different loci associated with TB BMD (i.e. variants in or close to *WNT4*, *WNT16/CPED1*, *RIN3*, *WLS*, *GALNT3*, *MEPE*, *LRP5*, *TNFSF11*). This analysis was performed within an investigation of genetic determinants of BMD following a life course approach involving 66,628 individuals, which identified over 80 loci explaining 10% of the TB BMD variability [17]. Age-stratified analyses revealed that genetic variants influencing BMD are mostly consistent across age groups. Only two out of these 80 loci displayed age-specific effects. The first association signal was mapped to *RIN3*, a gene with established influence on the development of Paget’s disease. The variant (MAF=0.12) showed evidence of a strong association with BMD in the age group 0–15 years with its effect decreasing with increasing age. Variants within, or, in close vicinity to *RIN3*, have not been pinpointed by GWAS of the femoral neck (FN) or lumbar spine (LS) BMD in adults. Yet, they have been suggestively associated with heel BMD derived from ultrasound measurements (eBMD) (*P* = 1.69 × 10^−7^) [22]. The second age-dependent signal was in the group aged 45–60 years in the 9q12 locus in the proximity of *TSHZ3*, but the high heterogeneity of the variant’s effect warrants further independent validation. Moreover, variants in the osteoprotegerin ligand (*RANKL*) and the *oestrogen receptor 1* (*ESR1*) showed significant evidence for age-dependent effects (Fig. 1a). Variants in *ESR1* displayed no association with BMD in individuals below the age of 15, with the majority of those consisting of prepubescent children. This lack of association was expected since the levels of estradiol before puberty are low [34].

Age heterogeneity has also been described in the effect of rs1800012 (*COLIA1*) on BMD. This variant, associated with fractures in postmenopausal women, exerts differential effects on BMD before and after puberty in girls [30]. Girls with homozygous risk alleles (TT) had decreased BMD before puberty and delayed pubescent bone accrual indicating that the variant’s association to BMD may be mediated by age of menarche. Indeed, MR studies indicate a causal association between age of menarche and decreased BMD [35]. The authors concluded that these differential effects could be related to spans of high/low bone turnover [30]. By modelling longitudinal bone acquisition across 11,000 bone scans from age 5 to age 20, Cousminer and colleagues described 40 loci associated with bone accrual [29]. However, only five of these loci (i.e. mapping within or in close vicinity to *SOX11*, *PALLD*, *ACO1*, *TERF2IP*, *TLE5*) replicated in an independent cohort.

Genetic determinants of BMD can also exert site-specific effects as a reflection of the intrinsic complexity of the skeletal system (Fig. 1b). A study dissecting paediatric dual-energy X-ray absorptiometry (DXA) TB BMD scans showed that heritability estimates differ across skeletal sites, being higher on the skull and lowest at the appendicular skeleton [25]. Overall, 13 loci (mapping to or in the vicinity of *EYA4*, *COLEC10*, *LGR4*, *LRP5*, *TNFRSF11A*, *RSPO3*, *TNFSF11*, *GALNT3*, *RIN3*, *FUBP3*, *PTHL*, *WNT16*, *WNT4*) were associated with the TB, skull, lower or upper limb BMD (Fig. 1b) [25]. Most of these loci have also been described as associated with FN or LS BMD prone to fracture skeletal sites in older adults [21]. Even a more complex level of genetic heterogeneity has been described in relation to paediatric bone. By following a sex-specific analysis of BMD at different skeletal sites, researchers from Philadelphia observed association signals mapping to *SPTB* only in girls at LS BMD, and *IZUMO3* only in boys at FN BMD [31]. Another two novel signals, at *RBFOX1* and *TPBL2* loci, were observed in both boys and girls, associated with LS BMD and radius BMD, respectively [31]. Also, following a candidate gene approach, the same authors reported sex-specific effects for a rare variant near *EN1* and a common variant near *SOX6* [32]. Such sex-specific findings in the autosomes remain intriguing and await replication in other paediatric cohorts.

Multiple association signals mapping to the 7q31.31 locus have been consistently reported in GWAS of BMD of paediatric populations [17, 25–28, 31] and adult populations [21, 22, 36, 37]. There is considerable evidence that either *WNT16* [38–42], *CPED1* [39], *ING3* [43] or *FAM3C* [44] are underlying the association signal. As conditional independent markers have been described in this locus (a phenomenon known as allelic heterogeneity) [25, 26], it is even likely that all these genes are key factors in bone biology, as supported by a recent transcriptome-wide association study [45].

The main disadvantage of DXA is its two-dimensional projection of a three-dimensional structure, which results in areal density, not representing a true volumetric BMD. In contrast, peripheral quantitative computed tomography (pQCT) captures three-dimensional measurements allowing the differentiation of trabecular and cortical bone. These pQCT assessments provide insight into the micro-architectural properties that determine bone strength at peripheral sites (radius, tibia). Paternoster and colleagues performed a GWAS meta-analysis on pQCT outcomes seeking to assess genetic factors influencing volumetric (v)BMD in young individuals before accruing peak bone mass [33]. *TNFSF11*, *ESR1*, *TNFRSF11B* and *EYA4* displayed associations with cortical vBMD, whereas variants mapping to *GREM2*/*FMN2* is associated with trabecular vBMD [33].

Overall, these studies indicate that the genetic variants pivotal to BMD in early life are central to BMD throughout an individual’s life course. This contention is supported by the good predictive performance of polygenic risk scores (PRS) derived from FN or LS BMD GWASs in adults [21] on prepubertal BMD [46], peripubertal BMD and bone accrual [47] in three different paediatric cohorts of multiple ethnicities. Briefly, a PRS is calculated by summing the effects of risk alleles associated with a trait, in this case, BMD, to generate an aggregated estimate of genetic risk in the form of a score. Moreover, a PRS built from variants associated with fracture risk in adults was found to be predictive of BMD in children [48]. Thus, genetic risk prediction could in theory be initiated already in early childhood to detect groups of children at high risk of fracture [49]. While lifestyle counselling to increase physical activity and ensure optimal nutrition, including adequate calcium and vitamin D intake during childhood, is widely encouraged, individuals genetically predisposed to lower BMD and increased fracture risk would benefit the most from such interventions [50].

## Monogenic Basis of Paediatric Fracture

Some rare Mendelian conditions leading to low BMD and/or low-energy fractures in children have been characterized [51, 52], and previous reviews have extensively addressed how to clinically approach children with bone fragility [51, 53, 54].

In the last decade, several gene discoveries in the field of primary childhood-onset bone fragility have been made thanks to the extensive use of massively parallel sequencing (MPS) in families with rare bone diseases [54]. Currently, over 35 monogenic diseases characterized by paediatric osteoporotic fractures have been identified [52, 55]. Each of these conditions has a Mendelian inheritance and is caused by a rare mutation with a large effect size affecting either a single allele (monoallelic) or both alleles (biallelic) of a gene pivotal in bone remodelling and/or bone homeostasis [56]. Several loci linked to paediatric BMD in GWASs, such as *SP7*, *TNFSF11* and *COL1A1*, are also involved in monogenic forms of skeletal fragility [56–59].

### Monogenic Forms of Childhood-Onset Skeletal Fragility

#### Osteogenesis Imperfecta (OI)

OI is the most common cause of osteoporotic fractures in the paediatric population, with a prevalence of 1/10,000 births [60]. OI patients typically present with growth delay, skeletal deformities, dentinogenesis imperfecta, blue/grey sclerae and hearing impairment, although wide variable expressions are present. The severity of OI varies from perinatally lethal forms to mild phenotypes characterized by an increased susceptibility to fractures [61]. This large variability in disease severity depends on the functional role of the affected gene, on the type of the pathogenic variant, and on the impact of this change on the protein function. However, disease severity varies also between patients with identical disease-causing variants, so it is likely that genetic modifiers also contribute to this phenotypic variability. Although the fracture rate in OI is very variable, a recent nationwide register study in Denmark evaluating the number of long-bone fractures in all those with a diagnosis of OI, found a 10-fold increased fracture rate in OI patients between 0 and 19 years of age as compared to the general population (fracture rate ratio 10.7) [62]. Typically in OI cases, some fractures already occur in utero and vertebral compression fractures are not uncommon since early childhood.

Rare mutations in 18 genes have been linked to OI (Table 1). Approximately 85% of OI cases are due to monoallelic mutations in one of two genes encoding the most abundant bone extracellular matrix (ECM) protein, type I collagen (*COL1A1* and *COL1A2*) [63]. Structural defects, mainly altering the Xaa-Yaa-Gly repeats within the triple-helical region of type I collagen, usually lead to more severe clinical consequences than mutations causing a reduced amount of protein [64]. The other 16 OI genes are involved in type I collagen folding/modification/processing, regulate osteoblast differentiation/function and control mineralization (Table 1).

**Table 1:** Genes currently linked to osteogenesis imperfecta

Gene	Encoded protein	Inheritance pattern	Phenotype MIM no.	Protein function
*COL1A1*	Collagen type I alpha 1 chain	AD	166200, 166210, 166220, 259420	Collagen synthesis and structure
*COL1A2*	Collagen type I alpha 2 Chain	AD	166210, 166220, 259420	Collagen synthesis and structure
*P3H1*	Prolyl 3-hydroxylase 1	AR	610915	Collagen biosynthesis, folding and assembly
*PPIB*	Peptidylprolyl isomerase B	AR	259440	Collagen post-translational modification
*CRTAP*	Cartilage-associated protein	AR	610682	Collagen post-translational modification
*FKBP10*	65 KDa FK506-binding protein	AR	610968	Collagen processing and crosslinking
*SERPINH1*	Heat shock protein 47	AR	613848	Collagen processing and crosslinking
*BMP1*	Bone morphogenetic protein 1	AR	614856	Collagen processing and crosslinking
*KDELR2*	KDEL endoplasmic reticulum protein retention receptor 2	AR	619131	Intracellular recycling of ER resident collagen chaperones
*TMEM38B*	Transmembrane protein 38B	AR	615066	Osteoblast differentiation and function
*SP7*	Osterix	AR	613849	Osteoblast differentiation and function
*WNT1*	Wnt family member 1	AR	615220	Osteoblast differentiation and function
*CREB3L1*	Old astrocyte specifically induced substance (OASIS)	AR	616229	Osteoblast differentiation and function
*MBTPS2*	Membrane-bound transcription factor peptidase, site 2 (S2P)	XLR	301014	Osteoblast differentiation and function
*SPARC*	Osteonectin	AR	616507	Osteoblast differentiation and function
*SERPINF1*	Pigment epithelium–derived factor (PEDF)	AR	613982	Bone mineralization
*IFITM5*	Bone-restricted interferon-induced transmembrane protein-like protein (BRIL)	AD	610967	Bone mineralization
*TENT5/FAM46A*	Terminal nucleotidyltransferase 5A	AR	617952	Unknown

*AD*, autosomal dominant; *AR*, autosomal recessive; *XLR*, X-linked recessive; *MIM*, Mendelian inheritance in Man; *NA*, not available. List of conditions from Mortier et al., 2019 [52]

Recently, biallelic mutations in *KDELR2* were identified in four families with progressively deforming OI [55]. *KDELR2* mutations disrupt the binding of KDELR2 with the collagen chaperon HSP47, which accumulates within the collagen fibrils thus disrupting them. Biallelic mutations in the gene encoding HSP47 (*SERPINH1*) and in another molecular chaperon, named *FKBP10*, are well-known causes of progressively deforming OI due to misfolding of the collagen triple helix (Table 1) [65, 66].

Monoallelic mutations in *IFITM5* and *SERPINF1*, encoding bone-restricted ifitm-like protein 5 and the pigment epithelium–derived factor, lead to bone hypermineralization [67, 68]. For *IFITM*5, two mutations mapping to the UTR region (c.-14C>T, c.-9C>A), resulting in an elongated protein, and one missense substitution (p.Ser40Leu) have been described [69–72]. Murine models harbouring the c.-14C>T mutation show disturbed mineralization [73, 74]. Patients carrying the mutation present with short to normal stature, very low to normal BMD and in 90% of the cases vertebral compression fractures [70].

Biallelic *SERPINF1* mutations affect the early steps of mineralization by leading to the formation of atypical collagen fibrils and increased osteoid volume. This feature is also present in *Serpinf1* knockout mice and results in severe OI [68, 75, 76].

Finally, among the six genes regulating the differentiation and function of osteoblasts (Table 1), *MBTPS2* and *SPARC* (secreted protein acidic and cysteine rich) are those that have been most recently linked to OI. In 2016, two X-linked recessive *MBTPS2* missense changes were identified in two multigenerational families with moderate to severe OI [77]. *MBTPS2* mutations impair the cleavage of OASIS, an important osteogenic transcription factor, leading to impaired osteoblast differentiation and defective collagen hydroxylation [77].

*SPARC* (secreted protein acidic and cysteine rich), also known as osteonectin, is a glycoprotein that binds several proteins of the ECM. Two missense substitutions affecting the Ca^2+^-binding domain of SPARC have been linked to severe OI [78, 79].

Since the majority of OI patients harbour mutations in type I collagen, sequencing of the *COL1A1* and *COL1A2* genes is usually recommended as the first diagnostic approach. DNA-based testing of other genes known to cause OI is subsequently carried out. Thanks to the widespread use of MPS, the success rate for OI diagnosis has significantly increased in the past decade.

#### Primary Childhood-Onset Osteoporosis

Childhood-onset osteoporosis is usually characterized by low BMD and low-energy fractures at a young age, but unlike OI, without skeletal deformities or extra-skeletal features. The criteria for childhood-onset osteoporosis are as follows: a BMD *Z*-score, less or equal to −2.0, and a significant fracture history (defined as two or more long-bone fractures before the age of 10 or three or more long-bone fractures before the age of 18) [51, 53]. Furthermore, the presence alone of one or more vertebral compression fractures, even with normal BMD levels, is indicative of paediatric osteoporosis [51, 53]. Although these genetic forms of osteoporosis present already during childhood or adolescence, some subjects are only diagnosed during adulthood. A potential explanation could be that sometimes the phenotype is not so severe and fractures are considered to be a consequence of the physically active lifestyle of a child. Moreover, the presence of protective genetic modifiers and favourable lifestyle factors might prevent the disease from manifesting during childhood. Currently, four monogenic forms of childhood-onset osteoporosis due to pathogenic variants in *SGMS2*, *PLS3*, *WNT1* and *LRP5* have been characterized; they are described below.

*Calvarial doughnut lesions with bone fragility syndrome* (MIM no. 126550) presents with low-energy peripheral and spinal fractures, abnormal bone structure with cortical thinning and defective bone mineralization. Monoallelic mutations in *SGMS2*, encoding *Sphingomyelin Synthase 2* (*SMS2*), are responsible for this condition [80, 81]. SMS2 catalyses the synthesis of sphingomyelin, a phospholipid that is abundantly present in the cell membrane and that is involved in cholesterol metabolism [82].

X-linked dominant mutations in *PLS3* have also been identified in families with osteoporosis and low-energy fractures, particularly in the vertebral bodies of the spine (MIM 300910) [83–85]. Although many patients with *PLS3* osteoporosis receive a diagnosis in adult life, these subjects usually sustain multiple peripheral and vertebral compression fractures already during childhood [83–85]. Most of the paediatric patients so far reported as having *PLS3* osteoporosis feature low BMD values. As *PLS3* is located on the X-chromosome, male patients are generally more severely affected than females [86]. A recent study suggests that osteocyte dysfunction might explain the disease [87]. Moreover, patients with *PLS3* osteoporosis have an increased expression of Dickkopf WNT signalling pathway inhibitor 1 (DKK1), an antagonist of the WNT-β-catenin signalling pathway [88], as well as an altered miRNA profile in serum [89].

*WNT1*, encoding the Wnt family member 1, is another important player in the WNT-β-catenin signalling pathway underlying osteoporosis [90, 91]. Monoallelic *WNT1* mutations cause defective modelling of long bones and low BMD in childhood, and recurrent vertebral fractures, kyphosis and severe kyphosis in adulthood (MIM no. 615221) [90, 92]. Biomarker analysis performed in serum of patients with WNT1 osteoporosis revealed increased levels of fibroblast growth factor 23 [88]—a bone-derived hormone—, and a disease-specific miRNA signature [93]. Biallelic *WNT1* mutations give rise to severe OI [90, 91, 94]. WNT ligands bind to the Frizzled receptor and the co-receptors LDL receptor-related protein 5 or 6 (*LRP5* or *LRP6*) [95]. Therefore, it is not surprising that *LRP5* mutations are also linked to monogenic bone fragility: heterozygous mutations are associated with osteoporosis (MIM no. 166710) [96] while homozygous mutations cause osteoporosis-pseudoglioma syndrome (MIM no. 259770) [97], a severe condition characterized by osteoporosis and congenital blindness. In addition to OI and childhood-onset osteoporosis, other primary diseases and syndromes displaying low BMD and low-energy fractures from childhood onwards are summarized in Table 2. Importantly, some monogenic conditions characterized by high bone mass or impaired bone mineralization, such as osteopetrosis and hypophosphatasia, can also lead to paediatric fractures (Table 2). Identification of the genetic cause of recurrent fractures in children is decisive to provide timely genetic counselling and define guidelines on how to best manage the condition.

**Table 2:** Other monogenic bone diseases and syndromes featuring childhood-onset bone fragility and/or increased susceptibility to fractures

Disease/syndrome	Phenotype MIM no.	Gene, inheritance	Encoded protein	Protein function
B3GAT3 deficiency	245600	B3GAT3, AR	Beta-1,3-glucuronyltransferase 3	Glycosaminoglycans biosynthesis
Bruck syndrome 1	259450	*FKBP10*, AR	65 kDa FK506-binding protein	Collagen processing and crosslinking
Bruck syndrome 2	609220	*PLOD2*, AR	Lysyl Hydroxylase 2	Collagen processing and crosslinking
Cole-Carpenter dysplasia 1	112240	*P4HB*, AD	Prolyl 4-hydroxylase subunit beta	Collagen post-translational modification
Cole-Carpenter dysplasia 2	616294	*SEC24D*, AR	SEC24 homolog D, COPII coat complex component	Vesicle trafficking
Cutis laxa, autosomal recessive form, type 2A	219200	*ATP6V0A2*, AR	ATPase H+ transporting V0 subunit A2	Acidification of diverse cellular components
Cutis laxa, autosomal recessive form, type 2B	612940	*PYCR1*, AR	Pyrroline-5-carboxylate reductase 1	Secretory pathway
Ehlers-Danlos syndrome, kyphoscoliotic type, 1	225400	*PLOD1*, AR	Lysyl hydroxylase 1	Collagen processing and crosslinking
Ehlers-Danlos syndrome, kyphoscoliotic type, 2	614557	*FKBP14*, AR	FK506-binding protein 14	Collagen processing
Familial expansile osteolysis	174810	*TNFRSF11A*, AD	Tumor necrosis factor receptor superfamily member 11A	Activation of NF-kappa B and MAPK8/JNK pathways
Geroderma osteodysplasticum	231070	*GORAB*, AR	Golgin, RAB6 interacting	Secretory pathway
Gnathodiaphyseal dysplasia	166260	*ANO5*, AD	Anoctamin 5	Control of muscle contraction and relaxation
Hajdu-Cheney syndrome	102500	*NOTCH2*, AD	Notch receptor 2	Bone remodelling and homeostasis
Hypophosphatasia, Odontohypophosphatasia	146300	*ALPL*, AD, AR	Alkaline phosphatase, tissue-nonspecific isozyme	Skeletal mineralization
Metaphyseal dysplasia with maxillary hypoplasia	156510	*RUNX2*, AD	RUNX family transcription factor 2	Osteoblast differentiation
Osteopetrosis	166600, 611490	*CLCN7*, AD, AR	Chloride voltage-gated channel 7	Lysosomal function and bone resorption
Osteopetrosis with renal tubular acidosis	259730	*CA2*, AR	Carbonic anhydrase 2	Bone resorption and osteoclast differentiation
Osteopetrosis, autosomal recessive 2	259710	*TNFSF11*, AR	Osteoprotegerin ligand	Osteoclast differentiation and activation
Paget’s disease, juvenile form	239000	*TNFRSF11B*, AR	Osteoprotegerin	Osteoclastogenesis
Pycnodysostosis	265800	*CTSK*, AR	Cathepsin K	Bone resorption
Short stature, optic nerve atrophy and Pelger-Huet anomaly	614800	*NBAS*, AR	NBAS subunit Of NRZ tethering complex	Golgi-to-ER transport
Singleton-Merten dysplasia 1	182250	*IFIH1*, AD	Interferon induced with helicase C domain 1	RNA helicase
Singleton-Merten dysplasia 2	616298	*DDX58*, AD	DExD/H-box helicase 58	RNA helicase
Spondylo-ocular syndrome	605882	*XYLT2*, AR	Xylosyltransferase 2	Biosynthesis of proteoglycans
Wiedemann-Rautenstrauch syndrome	264090	*POLR3A*, AR	RNA polymerase III subunit A	Synthesis of small RNAs

*AD*, autosomal dominant; *AR*, autosomal recessive; *MIM*, Mendelian inheritance in Man. List of conditions from Mortier et al., 2019 [52]

## The Interplay Between Monogenic Risk Variants and Polygenic Background

The causes of (paediatric) fracture are multifactorial including genetic and environmental influences, as well as their interactions. Regarding genetic factors, in a small subset of children, a rare monogenic variant is inherited that causes a large increase in the risk of recurrent fractures, by disrupting coding sequences in key bone pathways. However, in most children, genetic risk will be delineated by common variants with small effects in fracture risk, but which potentially can cumulatively have large effects. Indeed, recently, a landmark study showed that for some children experiencing multiple fractures, and to a great extent assumed to have an undefined monogenic disease, the clinical presentation was actually the consequence of an unusual accumulation of common variants associated with poor BMD, as assessed by genetically predicted heel ultrasound BMD (a lower score reflects a lower genetically predicted BMD and an increased risk of fracture) [98]. In children with recurrent fractures, the average PRS was 0.47 standard deviations (SD) lower than that in a population-based study (*P*=1.1 × 10^−5^), and in the children with suspected Mendelian osteoporosis, the score was even lower (−0.76 SD, *P*=5.3 × 10^−10^), indicating increased genetic risk of fracture. It is also expected that the severity of different skeletal pathologies originating from monogenic mutations would be substantially modified by the polygenic background, as already shown for other diseases [99].

## Future Directions for Genetic Research in Paediatric Populations for Fracture Risk

Optimization of genotyping and next-generation sequencing technologies have enabled the analysis of individual human genomes at accessible costs and within a reasonable timeframe, with the potential to advance further the study of the biological mechanisms underlying skeletal disease. These will offer promising new molecular targets for the development of pharmacotherapies, propose new indications to existing treatments (drug repurposing) and facilitate patient stratification [100] aimed at improving clinical care. Yet, researchers in the field of skeletal health face barriers to accomplishing these goals given the scarce availability of large-scale *-omic* data drawn in bone cells or tissue. Fortunately, initiatives as the musculoskeletal portal [101] or the GEMSTONE action (https://cost-gemstone.eu/) are starting to tackle these gaps.

Large population biobanks provide an opportunity to investigate efficiently the relationship between genes and disease. The vast available genotyping data from biobanks together with improved algorithms for imputation and analysis of GWAS have set a new paradigm in the genetics of complex traits. Similarly, the emergence of sequencing data from these studies and particularly from large case collections [102, 103] will undoubtedly impact the rare disease research community by refining our understanding of monogenic diseases. Allele frequency data from large exome and genome aggregation projects such as the Genome Aggregation Database (gnomAD) [103] are crucial to the interpretation of medical resequencing data (i.e. defining the pathogenic potential of a genetic variant). On top of this, up to one-fifth of loci identified by GWAS include a gene that is mutated in a corresponding single-gene disorder [104]. Studies resequencing GWAS-implicated genes have already efficiently identified genes for monogenic or familial forms of disease (i.e. diabetes, or inflammatory bowel disease) [105]. The same strategy could, for example, help with identifying genes with major roles in osteogenesis that are not yet implicated in monogenic forms of osteoporosis.

Despite these advances, fractures in children, as in adults, are a complex trait with a variable contribution of the environment and of hundreds of genetic factors, therefore, requiring very big sample sizes to overcome this intrinsically high heterogeneity. Yet, our understanding of the process of bone accrual could greatly benefit from insight derived from GWAS on refined bone phenotypes, such as pQCT, periosteal expansion or cortical density and thickness directly related to bone strength. Another trait that might be of interest is the trabecular bone score, evaluating indirectly the homogeneity of bone micro-architecture at the LS, which has been shown to provide complementary information to BMD in fracture risk assessment in adults [106].

Additionally, overall musculoskeletal health in children may be captured pretty early in life by diverse phenotypes. Age of walking has shown an association to bone strength in late adolescence, indicating that easily measurable phenotypes registered during early childhood may be strong predictors of overall bone health, and could highlight novel genetic variants associated with childhood bone development [107]. Indexes of bone strength, capturing biomechanical properties of bone, have also been developed based on DXA and pQCT parameters and assessed in children and adolescents [13, 108].

It is important also to note that bone-related GWASs have not captured the level of diversity that exists globally, as they have been predominantly based on individuals of European ancestry. Notwithstanding the higher fracture rates in European children as compared to that in children from other ethnicities [109, 110], GWAS efforts targeting other populations would allow the use of techniques as trans-ethnic mapping to identify true risk variants [111, 112]. Moreover, the differences in linkage disequilibrium across ethnicities will limit the accuracy of a PRS generated from mainly European populations to predict disease risk in other ethnicities [113]. For instance, a FN BMD PRS based on common genetic variants and derived from a GWAS meta-analysis in European adults was associated with TB BMD in children of three different ethnic ancestral groups, namely European, African and Asian. However, the strength of the association decreased in the non-European groups [46]. Despite this, the frequency of those alleles associated with increased BMD was systematically elevated in individuals of African ancestry as compared to the other groups [46], mirroring the observation from epidemiological studies showing that on average individuals from African background have higher BMD [46]. Future GWASs conducted in non-European populations will be key to improve genomic medicine. Particularly, African genomes, which harbour the highest genetic diversity and shortest stretches of linkage disequilibrium, are ideal when trying to identify causal variants (e.g. by using fine-mapping). Latino and Asians are also of particular interest as they are underrepresented in public datasets and demonstrate an increased risk of fractures.

Examining the genetic underpinnings of diseases presenting with secondary low BMD and fractures can further contribute to our understanding of paediatric fracture risk. Diseases as thalassaemia, Crohn’s and coeliac can underlie recurrent fractures in children and be overlooked by clinicians. Thalassaemias are genetic disorders characterized by decreased haemoglobin; despite our poor understanding of mechanisms leading to bone fragility in this disease, fracture prevalence in thalassaemia syndromes is already increased at a young age [114]. Crohn’s disease is associated with poor skeletal health, due to the direct effects of chronic inflammation, prolonged glucocorticoid use, poor nutrition, delayed puberty and low muscle mass [115]. Coeliac disease, caused by an adverse autoimmune reaction to dietary gluten, leads to malabsorption of calcium and vitamin D and inflammation resulting in low BMD and osteomalacia with increased fracture risk [116].

## Conclusion

Further understanding of the genetic predisposition to fracture in children, both monogenic and polygenic aetiology, aids health care professionals to improve risk assessment and refine intervention strategies. Peak bone mass acquisition is a pivotal process starting early in life that is under substantial genetic control. Unravelling the genetic determinants of bone accrual and paediatric fracture susceptibility will help to pinpoint strategies to maximize peak bone mass, delay osteoporosis onset and decrease fracture risk later in life.
